# Predictors of extinction risk in large tropical forest mammals: From global to local

**DOI:** 10.1126/sciadv.aeb7543

**Published:** 2026-07-15

**Authors:** Simon D. Schowanek, Douglas Sheil, Lydia Beaudrot, Pierre Dupont, Santiago Espinosa, Vittoria Estienne, Julia E. Fa, Jonas Geldmann, Patrick A. Jansen, Steig E. Johnson, Francesco Rovero, Fernanda Santos, Asunción Semper-Pascual, Andrea Fernanda Vallejo Vargas, Jorge A. Ahumada, Emmanuel Akampurira, Rajan Amin, Robert Bitariho, Adeline Fayolle, Davy Fonteyn, Ilaria Greco, Marcela Guimarães Moreira Lima, Matthew Scott Luskin, David Kenfack, Emanuel H. Martin, Eustrate Uzabaho, Cédric Vermeulen, Richard Bischof

**Affiliations:** ^1^ Faculty of Environmental Sciences and Natural Resource Management, Norwegian University of Life Sciences (NMBU), Ås, Norway.; ^2^ Forest Ecology and Management, Wageningen University and Research, Wageningen, Netherlands.; ^3^ Center for International Forestry Research (CIFOR), Kota Bogor, Indonesia.; ^4^ Department of Integrative Biology, Michigan State University, Michigan, USA.; ^5^ Ecology, Evolution, and Behavior Program, Michigan State University, Michigan, USA.; ^6^ Facultad de Ciencias, Universidad Autónoma de San Luis Potosí, San Luis Potosí, México.; ^7^ Facultad de Ciencias Exactas y Naturales, Pontificia Universidad Católica del Ecuador, Quito, Ecuador.; ^8^ Nouabalé-Ndoki National Park, Wildlife Conservation Society – Congo Program, Brazzaville, Republic of the Congo.; ^9^ Department of Natural Sciences, School of Science and the Environment, Manchester Metropolitan University, Manchester, UK.; ^10^ Center for Macroecology, Evolution and Climate, Globe Institute, University of Copenhagen, Copenhagen, Denmark.; ^11^ Wildlife Ecology and Conservation Group, Wageningen University & Research, Wageningen, Netherlands.; ^12^ Smithsonian Tropical Research Institute, Panamá, República de Panamá.; ^13^ Department of Biology, Utrecht University, Padualaan 8, Utrecht 3584 CH, Netherlands.; ^14^ Department of Anthropology & Archaeology, University of Calgary, Calgary, Canada.; ^15^ Department of Biology, University of Florence, Florence, Italy.; ^16^ MUSE – Science Museum, Trento, Italy.; ^17^ Biogeography of Conservation and Macroecology Laboratory, Institute of Biological Sciences, Universidade Federal do Pará, Pará, Brazil.; ^18^ Departamento de Mastozoologia, Museu Paraense Emílio Goeldi, Belém, Brasil.; ^19^ WildMon, Lewes, DE, USA.; ^20^ Uganda Wildlife Research and Training College, Kasese, Uganda.; ^21^ Institute of Tropical Forest Conservation, Mbarara University of Science and Technology, Mbarara, Uganda.; ^22^ Conservation Programmes, Zoological Society of London, London, UK.; ^23^ TERRA Teaching and Research Centre (Forest Is Life), University of Liège, Gembloux, Belgium.; ^24^ CIRAD, UPR Forêts et Sociétés, Campus International de Baillarguet, Montpellier, France.; ^25^ Forêts et Sociétés, Université de Montpellier, CIRAD, Montpellier, France.; ^26^ School of the Environment, University of Queensland, Brisbane, Australia.; ^27^ ForestGEO, Smithsonian Tropical Research Institute, Washington, DC, USA.; ^28^ College of African Wildlife Management, Mweka, Tanzania.; ^29^ International Gorilla Conservation Programme, Musanze, Rwanda.

## Abstract

Studies can only guide conservation if their findings are informative at the scales at which practitioners and policy-makers operate. Yet, it is rarely tested whether large-scale studies reach similar conclusions to the smaller-scale studies on which conservation traditionally relies. We examine whether predictors of extinction risk are consistent across global, regional, and local scales, for 210 tropical forest mammal species (≥1 kg) that existed during the last 130,000 years, in 64 tropical forests, across three biogeographical realms. We found consistent predictors of extinction risk (body mass, generation length, diet, brain volume, and scansoriality) when analyses differed only in their spatial resolution. However, predictors differed when analyses also varied in their temporal extent. Macroecological findings about extinction risk can, thus, inform conservation at smaller scales, but they risk misidentifying threatened species if differences in temporal extent are not recognized.

## Teaser

Studies predicting extinction risk come to different conclusions if their analyses use different temporal extents.

## INTRODUCTION

Humans are causing species extinctions at a rate unprecedented in recorded history (*1*, *2*). Conservationists aim to mitigate these extinctions through research (generating knowledge to guide practice and policy), policy (committing to a course of action), and practice (conducting actions on the ground). While practice and policy can happen at various spatial or temporal scales, they are primarily implemented at national, subnational, or local scales (e.g., inside country borders or protected areas), and over comparatively short timescales (e.g., months, years). In contrast, scientific studies that can inform conservation are conducted across various scales. While some studies use spatiotemporal scales similar to those encountered by policy-makers and practitioners, others use larger scales, for example, global or continental spatial scales or centennial to millennial timescales (*3*–*6*). Because of this scale mismatch, scientific research at large spatiotemporal scales is often perceived as irrelevant for practical conservation (*3*, *5*–*7*).

To ensure large-scale studies become relevant for conservation, their findings must be informative at the scales at which practitioners and policy-makers act (*3*, *4*). This requires that the patterns described by scientific studies are scale generalizable [i.e., findings from one scale can be applied at another (*8*)] or alternatively that we understand why patterns vary across scales. For example, if paleoecological studies show that, globally, larger-bodied species have been more vulnerable to extinction over millennial timescales (*9*), scale generalization would suggest that body size has similar effects in present-day situations encountered by conservationists [e.g, see Rovero *et al.* (*10*) who describe a loss of large-bodied mammals close to human activities inside several present-day protected tropical forests]. Yet, while scale generalization (or the absence thereof) is often assumed, such assumptions are rarely tested.

Comparing findings across scales is challenging because trade-offs often exist between study resolution or grain (the smallest unit of observation) and extent [the spatial or temporal range covered (*8*)]. Small-scale studies offer high resolution but lack the spatial or temporal coverage to permit more general conclusions. Large-scale studies, on the other hand, such as macroecological or paleoecological studies, cover broader extents but typically lack the spatial or temporal resolution needed to describe patterns in detail, which are needed for implementing local conservation practices. However, advances in automated monitoring methods, like camera trapping, increasingly offer datasets with extensive geographical coverage and high data resolution (*11*). Although such datasets remain biased toward taxa and sites of high conservation interest (e.g., protected areas) (*12*, *13*), they can provide extensive and detailed views of ecological patterns that may help bridge the gap between research at large scales and application at smaller scales.

Predicting species’ extinction risk at different scales offers an excellent means to test the generalizability of findings across these scales. First, preventing extinction is at the heart of conservation and studies of extinction are conducted on several spatiotemporal scales, ranging from global [e.g., (*14*)], to regional [e.g., (*15*)], to local [e.g., (*16*)]. However, some have questioned the usefulness of large-scale studies of extinction risk, arguing that the findings of such studies are often inconsistent and have led to few clear and actionable conservation messages (*17*). Conversely, local, and short-term studies are more easily affected by shifting baselines (*18*), which can obscure ecological and evolutionary patterns (*19*, *20*).

Second, extinction is a useful study system because, in theory, species losses at different scales are causally connected. A species’ extinction at large scales results from local extinctions throughout its range, suggesting that the patterns and predictors of extinction should, to some degree, be comparable across scales. Nonetheless, several social, demographic, and ecological patterns are scale-variant, meaning that the same phenomenon can effectively be treated as independent phenomena when analyzed at different scales (*8*). For example, while a species’ global range comprises the space use of all individuals, the space use of any given individual may teach us little about a species’ global range, and vice versa. Understanding which patterns are scale-variant and which are not will help improve the practical utility of large-scale studies in informing local conservation.

**Fig. 1.**
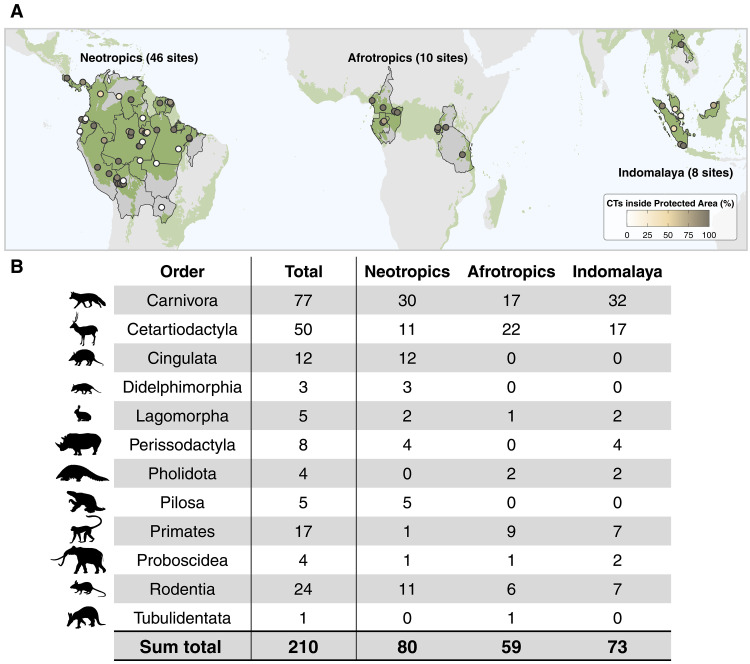
Biogeographic realms, regions, and sites and the tropical forest mammals studied. (**A**) The locations of the 64 sites used in this study, as well as the regions in which they are located (highlighted areas). Regions are approximately evenly-sized areas that broadly share a biogeographical history, and fall under the same administrative entities (see Materials and Methods). The color of the points shows how many of the sites’ camera traps are located inside a protected area. The green layer shows the distribution of tropical forest. (**B**) A summary of the species used in this study, summarized by order and biogeographical realm. Note that some species occur in multiple realms.

Here, we test whether extrinsic and intrinsic predictors of extinction risk in medium to large (≥1 kg, detectable by camera traps) tropical forest mammals are generalizable across global, regional, and local scales. Using a Bayesian framework, we model the survival probability (the inverse of extinction probability) of 210 species in 64 tropical rainforest sites (sampled inside protected areas, *n* = 31; partially inside protected areas, *n* = 25; and outside protected areas, *n* = 8) across three biogeographical realms at these three scales (Fig. 1). The model consists of three logistic regressions, representing studies of survival at the different scales. The global regression describes which species survived across the tropics since the Eemian period, which started 130,000 years BP, providing a macroecological comparison with limited human impacts. The regional regression describes which species have persisted within a region. Regions are defined as similarly sized areas that broadly share a biogeographical and administrative history (see Materials and Methods). Most regions correspond to countries and resemble the administrative entities with which national and international conservation policy is concerned. The local regression describes which species have persisted at a given study site (e.g., a protected area).

**Fig. 2.**
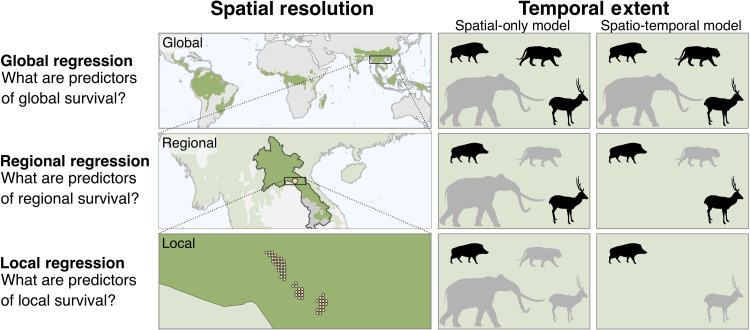
Framework used to test the similarity of predictors of species’ extinction risk across spatial and temporal scales. Column one describes the three regressions used in each model. Column two shows the spatial resolution of each regression. Columns three and four represent the species pools at different scales for the spatial-only and the spatiotemporal models, respectively. Black silhouettes represent extant species. Gray silhouettes represent extinct species. The spatial-only model changes the spatial resolution from global to regional to local but the temporal extent remains identical for each regression. In contrast, the spatiotemporal model changes the spatial resolution and the temporal extent. It sequentially removes extinct species resulting in nested species pools for each regression.

To compare how extinction risk predictors vary over space and time, we run two versions of this model (Fig. 2). In the spatial-only model, the only difference between regressions is their spatial resolution. The species pool is the same at each scale, though fewer species survive at smaller scales. In the spatiotemporal model, the spatial resolution changes as well as the temporal extent. We do so by excluding species from the species pool at smaller scales if they have already gone extinct at a higher scale. This gives each regression different but nested species pools. Excluding these species broadly corresponds to the chronology of mammal extinctions and the subsequent absence of species from scientific studies due to shifting baselines (*18*). In the spatiotemporal model, the global, regional and local scales, therefore, correspond to studies with long, intermediate and short temporal extents, respectively. In both models, the regressions have the same pantropical extent.

We determined species’ presences and absences by combining present-natural ranges from PHYLACINE version 1.2.1 [estimates of where species would live in the absence of human impacts (*21*)], ranges from the International Union for Conservation of Nature (IUCN), and camera trap observations (see Materials and Methods). We included three types of predictor variables: biogeographical variables (biogeographical realm), species traits [body mass (*21*), relative brain volume (*22*, *23*), relative generation length (*24*), vertebrate carnivory (*21*), and scansoriality, i.e., a species’ ability to climb (*25*, *26*)], and site variables [forest cover (*27*), human population density (*28*), and protected area coverage (*29*)]. We included site variables in the local regression only to account for their known effects on extinction risk rather than to test their consistency across scales. We also ran an alternative version of both models, which included phylogenetic variables, accounting for relatedness between species (see Materials and Methods). We used a reversible-jump Markov Chain Monte Carlo (MCMC) approach to quantify the probability that a predictor should be included in a regression (*30*). The resulting inclusion probability provides a metric of the evidential support for each predictor (see Materials and Methods). We considered variables significant if they were more likely than not to be included in a regression (inclusion probability >0.5).

## RESULTS

### Spatial-only model

**Fig. 3.**
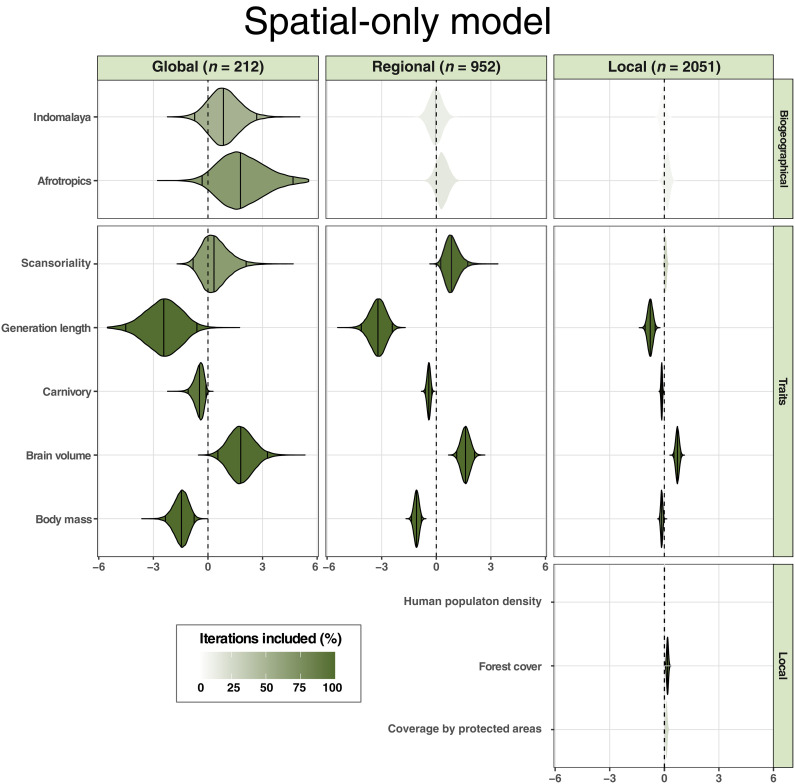
Predictors of extinction risk were similar across the global, regional, and local scales when regressions only varied in their spatial resolution. Violin plots show posterior distributions of coefficient estimates of the spatial-only model. Vertical lines inside the violin plots show the 95% credible intervals and median values. The color intensity indicates how often each predictor was included in the model. Variables included in more than 50% of the iterations are drawn with black borders. Variables included in fewer than 50% of the iterations are drawn without black borders. The Neotropics are the intercept level to which the other biogeographical realms are compared.

In the spatial-only model, species’ traits were strong predictors of survival probability and were consistent in direction across global, regional and local spatial scales (Fig. 3 and tables S1 to S3). Extinction risk was higher for species with large body masses, small brains, carnivorous diets, long generation lengths, or terrestrial (rather than scansorial) habits. Effect sizes were smaller at the local scale than at the regional and global scale. Scansoriality was even nonsignificant at the local scale, although its direction, scansorial species being less vulnerable than terrestrial species, was consistent with those at the global and regional scales.

Species survival probabilities also varied depending on where species occurred. First, species from different biogeographical realms had different survival probabilities on the global scale; Afrotropical and Indomalayan species had higher survival probabilities than Neotropical species, with the highest survival in the Afrotropics. Second, at the local scale, species were more likely to survive in areas with a high forest cover, whereas human population density or protection status of sites did not significantly influence survival probabilities.

### Spatiotemporal model

**Fig. 4.**
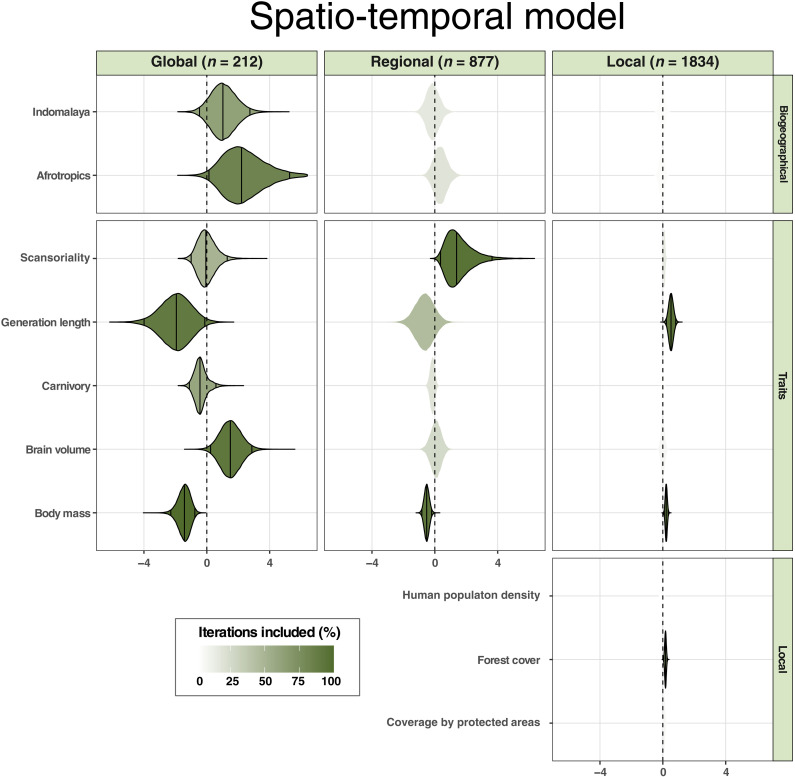
Predictors of extinction risk varied across the global, regional, and local scales when regressions vary in their spatial resolution and temporal extent. Violin plots show posterior distributions of coefficient estimates of the spatiotemporal model. Vertical lines inside the violin plots show the 95% credible intervals and median values. The color intensity indicates how often each predictor was included in the model. Variables included in more than 50% of the iterations are drawn with black borders. Variables included in fewer than 50% of the iterations are drawn without black borders. The Neotropics are the intercept level to which the other biogeographical realms are compared.

In the spatiotemporal model, predictors of extinction risk were not consistent across scales (Fig. 4 and tables S4 to S6). At each scale, predictors differed in direction and magnitude. At the global, long-term scale, results resembled the spatial-only model; extinction was more likely for species that were large, had long generation lengths, small brains, or carnivorous diets. At the regional scale, small species and scansorial species were more likely to survive, but no other traits were significant. However, at the local, short-term scale, large species and species with long generation lengths were more likely to survive, opposite to findings at the global, long-term scale.

Biogeographical differences in survival probability were similar to those in the spatial-only model. At the global scale, Afrotropical and Indomalayan species had higher survival probabilities than Neotropical species, and the effect was strongest in the Afrotropics. Likewise, at the local scale, species were more likely to survive in areas with higher forest cover.

### Phylogenetic models

We also ran both models with a correction accounting for the phylogenetic relationships between species (see Materials and Methods and Supplementary Materials Models with Phylogenetic Correction). In both cases, phylogeny was a significant predictor of survival probability at all three scales (figs. S1 and S2). However, adding a phylogenetic correction to the model did not meaningfully change the effect of other predictors, except for carnivory which was strongly correlated with phylogeny (fig. S6).

## DISCUSSION

For large-scale studies to inform conservation, their findings must be relevant at the often narrower spatial and temporal scales at which practitioners and policy-makers work and at scales that are ecologically relevant for species (*4*). We studied the degree to which the predictors of extinction risk were consistent across scales for tropical forest mammals. We found that intrinsic predictors of extinction risk were similar across the global, regional, and local scale when regressions only varied in their spatial resolution and used the same 130,000-year temporal extent (spatial-only model). Under this model, mammal species with longer generations, carnivorous diets, smaller brains, or larger bodies were more likely to go extinct at all spatial scales. In contrast, extinction risk predictors were variable when the temporal extents of the regressions became increasingly shorter (spatiotemporal model). While the spatiotemporal model identified similar predictors as the spatial-only model at the global scale, it found little consistency between large versus small spatial scales, even finding opposite effects in the case of body mass and generation length. This discrepancy between scales and models suggests that the processes driving extinction at the local scale today may differ from those that have been driving large-mammal extinctions over the past 130,000 years.

Moreover, these contrasting results highlight how different temporal extents provide different perspectives on these changing drivers of extinction. Large-scale extinction risk studies have been criticized for their inconsistent findings and their lack of clear conservation messages (*17*). Our results suggest that this variation amongst study findings may partly arise due to differences in their temporal extent, which may lead to opposite conclusions. For example, studies of brain size and its effect on extinction risk that only include extant species have found that extinction risk is higher for species with large brains (*31*, *32*). In contrast, studies that also include globally extinct species, including taxa from the Pleistocene or earlier periods, found that extinction risk is lower for species with large brains (*22*, *33*). Nonetheless, the temporal extent of studies often remains implicit, and has been less examined than the spatial and taxonomic extent [for example, see Cardillo and Meijaard (*17*) who discuss the influence of spatial and taxonomic but not temporal extents on studies of extinction risk].

Temporal extents change the observable ecological patterns in studies of extinction because they affect the species pool (and thereby also taxonomic extent). By only considering recent temporal extents, which can happen due to shifting baselines (*18*), studies of extinction exclude species that went extinct previously. However, because anthropogenic extinctions are nonrandom, such species are more likely to have extreme trait values, such as large body sizes or unique diets (*9*, *34*). The surviving species pool will, therefore, have properties that differ from those of the original species pool, and inferences based on one may not apply to the other (*35*). Specifically, studies with short temporal extents view a more limited range of the possible set of trait values, which can dampen the observable effect of processes that caused extinction in the past. Short temporal extents can, therefore, hide long-term drivers of extinction or underestimate their importance.

The reverse may also be true. Including long-extinct species can overshadow contemporary trends affecting the remaining species, either because vulnerable species have been removed or because the drivers of extinction have changed. For example, our spatiotemporal model predicted that long-lived, large-bodied species were more likely to survive at the local, short-term scale. We hypothesize this pattern exists because camera trap data (including those in our study) predominantly come from protected areas (*12*, *36*); these are sites where threatened species receive special protection and where large-mammal population trends tend to be more positive (*36*). We did not detect similar effects in the spatial-only model. The strong negative effects that body size and generation length have historically had on extinction likely overrode the more subtle contemporary correlation between large mammals and survival in protected areas.

Conservationists can, thus, use macroecological findings to identify threatened species if they are aware of the extent of the studies they rely on (temporal or otherwise). However, uncritically adopting macroecological findings can cause one to misidentify the determinants of extinction risk at smaller scales. Long-term extents will give greater emphasis to trait values that have already been lost, which can be valuable when highlighting the functional gaps in assemblages and guiding functional restoration efforts. Yet, emphasizing extinction predictors that are no longer relevant may prevent conservationists from accurately identifying present-day threatened species. Studies with short temporal extents are, arguably, better at identifying present-day risks. However, if they exclude long-extinct species, some predictors of extinction may also be missed. For example, imagine an extinction driver is ongoing but most species vulnerable to the driver have perished and are forgotten. Yet, a few species vulnerable to the driver survived in the community (e.g., due to random chance). Such species risk being classified as “not vulnerable” because there is no recent evidence that their traits predispose them to extinction. Analyzing extinction risk from both short and long-term perspectives may, ultimately, provide the most comprehensive view of extinction risk, as it can highlight changes in the relative importance of different drivers of extinction.

### Do our findings apply to other taxa, ecosystems, or predictors?

The findings of our spatial-only model concur with well-known macroecological patterns of extinction in vertebrates, such as the fact that species with large body sizes, long generation lengths and comparatively small brains have a higher extinction risk (*14*, *22*, *37*, *38*). Likewise, we identified the Afrotropics as having the fewest global extinctions, followed by Indomalaya and lastly, the Neotropics, likely reflecting species’ adaptation or coevolution with humans in areas that have a long history of human presence (*39*). As reported in previous studies, our results also suggest that carnivores are comparatively more threatened than other dietary groups [(*40*); but see (*41*)]. Because these patterns are consistent with studies on other taxa and habitats, we suspect our findings will apply to vertebrates more broadly, and not just terrestrial forest mammals.

Scale generalizability may not be equal for all processes, however. We studied a process with clear links between scales, where global extinctions result directly from extinctions at smaller scales. Scale generalizability may be lower in other processes. For example, research suggests species’ habitat selection may be difficult to generalize, because the act of selecting habitats happens on one or only a few scales. Certain scales may, therefore, be more informative than others (*42*).

In addition, scale generalizability may be lower for extrinsic predictors of extinction risk. Our study primarily compared similarities between species’ trait variables, but extrinsic variables, such as forest cover, economic status, or cultural attitudes are equally important for conservation issues. It is, arguably, unlikely that all extrinsic predictors would exhibit similar patterns across scales. The one extrinsic variable we compared across scales (biogeography) did not display scale generalizability.

Last, our data predominantly come from protected areas, reflecting available camera trap data (*12*). This could influence the extinction predictors we identify. Nonprotected areas often experience pressures that are more intense or unlike those found in protected areas, such as agricultural expansion, urbanization, hunting, or resource extraction (*43*). Consequently, it is unclear whether the positive trends in the spatiotemporal model’s local regression are the result of scale differences or of the data at our disposal. This uncertainty does not change our conclusion, however, because the contrasting findings of both models still illustrate how changes in temporal extent can change a study’s findings. Nevertheless, the higher survival of species with traits that are normally associated with extinction risk does raise questions about the influence of protected areas on biodiversity data. Possibly, such findings indicate that protected areas can safeguard vulnerable species, as was suggested for species with large body sizes (*36*). Yet, such patterns could also arise due to selection biases in protected area placement or extinction debts (*43*).

More studies are, therefore, needed, that analyze to what degree studies with large spatial or temporal extents can inform conservation. Ideally, such studies should analyze different processes, different types of predictors. For example, local scale variables could be expanded to include accessibility to people (e.g., proximity to roads) and compared with similar data at regional scales. In addition, analyses could include different study sites (including nonprotected areas), and diverse taxa. We specifically call for more research regarding the effect of large temporal extents on local-scale studies. Many conservation actions take place at the local scale, but we found that temporal extents could fundamentally change the conclusions of local-scale studies on which such actions often rely. Ultimately, we hope that such studies will help clarify which macroecological findings are relevant for more local conservation efforts and guide more effective conservation strategies.

## MATERIALS AND METHODS

### Site selection

We assembled a comprehensive list of camera trap locations (*n* = 337) across the pantropics, situated inside wet tropical forests of varying protection status. We collated data from the TEAM camera trapping protocol (*44*), Amazonia CAMTRAP (*45*), as well as individual datasets provided by authors of this manuscript [e.g., (*46*)]. First, we manually eliminated datasets outside forest areas (*n* = 3). We also removed a single site on Madagascar. Madagascar is biogeographically distinct and we did not want to introduce sampling bias by representing this region with a single site.

To eliminate duplicate records and minimize spatial autocorrelation among sites, we calculated 10 km buffers around each camera trap. Camera traps with overlapping buffers were then clustered together, resulting in 140 camera trap clusters (henceforth, sites) that were at least 20 km apart. We visually inspected these clusters to ensure they were clearly separated.

The detection of species is not constant between cameras and between sites (*47*). Therefore, we assessed the species accumulation curve at each site, retaining only sites where the curve converged to a slope of 0.2 or lower (indicating that most species present at the site and detectable by camera traps had been detected; see fig. S3). This process yielded a final selection of 64 sites, 46 in the Neotropical realm, 10 in the Afrotropical realm, and 8 in the Indomalayan realm. Because of the clustering methodology, our study sites could be entirely within, partially inside or outside protected areas (as defined by the World Database on Protected Areas version 1.6 October 2023). However, sites that are either partially or fully within protected areas are more common in our dataset (*n* = 56; fig. S4), as camera traps are still primarily deployed in protected areas and other areas of conservation interest (*12*, *36*).

### Species lists

For each site, we created four species lists: (i) present-natural, (ii) surviving globally, (iii) surviving regionally, and (iv) surviving locally (fig. S5). These species lists are nested: If a species occurs in a given species list it also occurs in the species lists above it. We used the different species lists to create the presence-absences used in the model regressions (see the “Statistical model” section).

The present-natural species list is the total species pool. It is based on the species list of PHYLACINE version 1.2.1 (*21*) rather than camera-trap data, and contains all species expected to occur at a site if there had been no local or global anthropogenic extinctions since the Eemian period, which started ±130,000 years ago. The Eemian period provides a reference for a world with a comparable climate and species composition but with limited human impacts and is commonly used in macroecological studies of extinction [e.g., (*19*)]. We created the present-natural species lists by overlaying the present-natural ranges from PHYLACINE version 1.2.1 (*21*) with the locations of each site.

Present-natural ranges are not past ranges. They estimate where species would occur today in the absence of human impacts. For most species (*n* = 156), these ranges are identical to their IUCN ranges. For species with human-caused range modifications (*n* = 54, including global extinctions), the present-natural ranges predict the distribution without anthropogenic modifications (*21*). These modified ranges were estimated using a variety of methods [see (*21*)]. For extant species, these mostly consisted of reversing known or suspected anthropogenic range changes, merging disjunct ranges, or extending the species’ range to include contiguous, suitable habitat. For globally extinct species, the present-natural range was most commonly estimated based on the present-day occurrence of species with which the extinct species coexisted: An area was considered suitable for the extinct species today, if it contained at least 50% of the extant species with which the extinct species to co-occurred in the (sub)fossil record [for details, see (*21*)].

To keep the present-natural species list comparable to the surviving locally species lists, which is based on camera trap data (see below), we excluded species with primarily aquatic or arboreal lifestyles (although we retained scansorial species, i.e., largely terrestrial species with the ability to climb), as well as species smaller than 1 kg, because they are difficult to detect with ground-based camera traps (*48*). We also excluded extinct human species because their interactions with *Homo sapiens* were likely different from those of nonhuman mammals and because conservation research does not generally study other human species.

Because of the coarse resolution of present-natural maps (96.5 by 96.5 km at 30° North and South), our species list included species that would not have occurred in the tropical rainforests of our study sites. Therefore, we checked the ecology of each remaining species and excluded species that would not occur in tropical rainforests (*n* = 260, see Supplementary Materials SI_Data_2_excluded_species.csv). We relied on the handbook of the mammals of the world series where possible (*49*–*52*), and otherwise on other scientific (e.g., herbitraits) and gray literature (e.g., IUCN red list). We paid particular attention to globally extinct species, and specifically excluded grazers and species tied to open ecosystems.

There were 57 (of 2051) cases where camera traps recorded extant species at a site even though they were not predicted to occur there by PHYLACINE’s present-natural maps. We added these species to all relevant species lists and treated them as if they had been predicted to occur there. Most of these inconsistencies were the result of taxonomic differences between datasets or IUCN ranges that underestimated species’ ranges and were subsequently copied by PHYLACINE (Supplementary Materials SI_Data_3_range_inconsistencies.csv).

Because of uncertainties in the (sub)fossil record, it is possible that some undocumented extinctions have occurred over the last 130,000 years, especially in tropical forests which do not preserve fossils well (*53*). Some globally extinct species may, therefore, still be missing from our present-natural species pool. Nonetheless, the extinctions of Quaternary medium-large mammals are well-known compared to most other species groups and periods, due to its recency and the fact that mammals are among the most-studied groups in paleo-archaeological sciences (*54*).

The surviving globally species list for a given site contains all species from the sites’ present-natural species list that remain globally extant. Of the 210 species, 199 remain globally extant and 11 have gone globally extinct.

The surviving regionally species list for a given site contains all species from the sites’ surviving globally species list that are still extant in the region in which the site is located (Fig. 1). We created the surviving regionally species list by overlaying IUCN ranges (version 2022-1, using the extant, probably extant, possibly extant parts of the range) with the “basic recording units” from the World Geographical Scheme for Recording Plant Distributions (*55*). Basic recording units (which we call “regions”) are approximately evenly sized geographical entities delineated by political borders and coastlines. They represent areas that broadly share a biogeographical history and fall under the same administrative entities, meaning they are subject to similar conservation policies. As such, they better reflect political realities of national and international conservation policy than maps of traditional ecoregions.

In the Virunga Massive, the camera trap setup straddled the border between Rwanda and Uganda, thus overlapping with two basic recording units. However, we assigned Rwanda as the basic recording unit as 55 of 60 camera traps were located in Rwanda and as all species recorded on the Ugandan site were also recorded on the Rwandan side. Species were considered surviving in the state if their IUCN range maps overlapped with the basic recording units in which the site was located.

The surviving locally species list contains all species extant at a site. We listed species as locally surviving if they had been observed at the site according to our camera trap records. We removed all records of humans, domestic species, and species smaller than 1 kg. Moreover, we removed records of primarily aquatic or arboreal species (although we retained scansorial species). Removing these species left us with 142 species that were observed at one or more of our sites.

Note that our method assumes that a species should occur at a site if that site falls within the species’ present-natural range. However, species’ ranges are seldom completely occupied, and while likely, we cannot be certain that a given “predicted but absent” species would have occurred at any given site. The local scale “extinctions” in our study are therefore, “presumed extinctions,” which potentially inflates the number of “extinct species” at the local scale.

### Predictor variables

We collected several potential predictors of extinction and divided them into the following groups: biogeographical variables, species traits, site variables, and phylogenetic variables. Biogeographical variables describe biogeographical differences between regions. Species traits describe properties of species (e.g., body mass). Site variables describe properties of the site (e.g., forest cover). Phylogenetic variables describe the phylogenetic relationships between species.

#### Biogeographical variables

To account for biogeographical differences in extinction risk, we assigned each site to one of three realms with unique biogeographical histories: the Afrotropics, Indomalaya, and the Neotropics. We included the biogeographical realm as a potential predictor of extinction at each scale, using the Neotropics as the reference. It accounts for differences in extinction risk between biogeographical areas that we cannot explain with our other variables.

#### Species traits

For all 210 species in our study, we collected trait information on: body mass (kg), vertebrate carnivory (%), scansoriality (terrestrial | scansorial), generation length (days), and brain endocast volume (ml). All these traits have been suggested as predictors of extinction by other studies (*22*, *37*, *56*, *57*). Body mass is a proxy for body size, one of the most fundamental correlates of a species life history (*58*). Vertebrate carnivory reflects a species diet and trophic level. Here, it is defined as the percentage of vertebrate consumption in the diet, as recorded by PHYLACINE version 1.2.1 (*21*). Because of how it is defined, this variable is almost entirely the inverse of a species’ plant consumption, and thus allows us to differentiate between herbivores, omnivores, and carnivores. Scansoriality reflects a species’ arboreality, and thus its ability to avoid ground-based human hunters, and to some extent its dependence on tree cover (*37*). Generation length reflects species demographic rates, and correlates with how quickly species can recover from low population sizes. Brain volume affects extinction risk, as large (relative) brain volumes give species the behavioral flexibility to deal with threats and novel environments (*22*, *33*, *59*), but it also requires large energetic allocations that could increase extinction risk (*31*, *32*).

We collected body mass and diet estimates from PHYLACINE (*21*). We collected scansoriality from Lundgren *et al.* (*25*) and Wilman *et al.* (*26*) and completed data gaps by manually reviewing the ecology of species with missing arboreality data. We collected endocast volumes from Dembitzer *et al.* (*22*) or derived them from brain mass estimates in Burger *et al.* (*23*). We converted brain mass to endocast volume, following the method in (*22*). First, we converted brain mass to brain volume by dividing it by 1.036. Next, we converted brain volume to endocast volume using the formula: log(Endocast Volume) = −0.0015 + 1.0222 * log(Brain Volume). We collected generation length from Pacifici *et al.* (*24*). We phylogenetically imputed missing traits (table S7) using the PHYLACINE version 1.2.1 phylogenies, and the Rphylopars package (*60*) in R version 4.2.2 (2022-10-31). We imputed all missing traits of all nonflying, terrestrial mammals (*n* = 4535 species) using 1000 different phylogenetic trees, and using mass, diet, scansoriality, endocast volume, and generation length as predictors. We, thus, had 1000 imputed estimates for each missing trait value, which were later provided to the model (see the “Statistical model” section).

To emphasize the relative size differences between species we used the logarithm of body mass rather than absolute body mass values in our analysis. To avoid multicollinearity between body mass, brain volume, and generation length, we regressed log-transformed body mass against log-transformed brain volume and against log-transformed generation length using a simple linear model. We used the residuals of these regressions as input variables in our statistical model. These residuals model the effects of brain volume and generation length on extinction risk that cannot be explained by body mass. The subsequent collinearity between variables is reported in figs. S6 and S7.

#### Site variables

Our model included three site variables: degree of forest cover, human population density, and the percentage of camera traps at a site located inside protected areas. We estimated forest cover by using the global forest change (GFC) dataset (*27*), which contains canopy cover data for the year 2000 (30 by 30 m resolution), as well as data on net changes in canopy cover between 2000 and 2012. First, we generated a forest cover map for the year 2000 (defining forest as tree canopy cover >75%, and masking water bodies). Next, we calculated the net change from 2000 to 2012 and used it to create forest cover maps for the year 2012. We then calculated the forest cover in 2012 in a 5, 10, and 20 km buffer around each site. We extracted population density from CIESIN’s Gridded Population of the World (version 4.11) dataset (*28*). This dataset maps human population density across the planet (30–arc sec resolution) in 5-year time steps from 2000 until 2020. We calculated the median human population density in 2010 in a 5, 10, and 20 km buffer around each site. We log-transformed the data before putting it in the model. The main models used the 10 km buffer, but to ensure that model findings were stable, we also ran models using the 5 and 20 km buffers. These did not meaningfully change the results (figs. S8 to S11).

#### Phylogenetic variables

We ran alternative versions of each model (Supplementary Materials Models with Phylogenetic Correction), using phylogenetic eigenvectors to account for the phylogenetic relationships among species (*61*). We used Rphylopars (*60*) to calculate the phylogenetic distances between all species based on 1000 phylogenetic trees from PHYLACINE (*21*). We performed a principal coordinate analysis (PCoA) on each of the resulting distance matrices. We averaged the 1000 PCoA outputs and included the first two axes of the averaged output in our model as explanatory variables, which explained 39.19% of variation.

### Statistical model

We developed a Bayesian model to assess the survival probability of all 210 mammal species (see Vignette.html in the Supplementary Materials) across all 64 sites (Fig. 1) and across three different scales (global, regional, and local). The Bayesian models consisted of three logistic regressions, corresponding to each scale (Fig. 2). The global regression modeled the variables that predict whether species survive globally. The regional regression modeled the variables that predict whether species survive regionally. The local regression modeled the variables that predict whether species survive locally.

The survival probability of species sp at scale sc can be expressed as the outcome of a regression:

$$\text{Logit}\left(\Psi_{\text{sp},\text{sc}}\right)=\beta_{0}+I_{1}\;\beta_{1}\;x_{1}+\ldots +I_{n}\;\beta_{n}\;x_{n}$$

where β_0_ is the intercept, β_1*-n*_ are the coefficients of predictor *i*, and *x_i_* is the value of predictor *i*. Indicator variables *I_i_* denote inclusion (*I_i_* = 1) or omission (*I_i_* = 0) of a given coefficient and associated predictor in the regression. The inclusion variables are sampled from a Bernoulli distribution with inclusion probability parameter *p_i_*

$$I_{i}∼\text{dbern}\left(p_{i}\right)$$

Parameter inclusion probability estimation is implemented as part of reversible jump MCMC sampling (*rj MCMC*) to allow sampling of the entire parameter space and proper model convergence.

The presence of species *sp* at scale *sc* (*Presence*_*sp,sc*_) is then estimated using a Bernoulli distribution.

$${\text{Presence}}_{\text{sp},\text{sc}}∼\text{dbern}\left(\Psi_{\text{sp},\text{sc}}\right)$$

We ran two different versions of the model (Fig. 2). In the spatial-only model, the only difference between regressions is their spatial resolution (Table 1). In the spatiotemporal model, the spatial resolution changes as well as the temporal extent (Table 2). We do so by excluding species from smaller scales if they have already gone extinct at a higher scale, broadly corresponding to the chronology of mammal extinctions and the subsequent absence of species from scientific studies due to shifting baselines (*17*). In the spatiotemporal model, the global, regional, and local scales, therefore, correspond to studies with long, intermediate, and short temporal extents, respectively. Excluding these species gives each regression different but nested species pools. Note that both models are identical at the global scale and that all regressions have the same pantropical extent.

**Table 1.** The spatial-only model and the species lists they use in each regression.

	Total species pool	Present species
Global	Present-natural	Surviving globally
Regional	Present-natural	Surviving regionally
Local	Present-natural	Surviving locally

**Table 2.** The spatiotemporal model and the species lists they use in each regression.

	Total species pool	Present species
Global	Present-natural	Surviving globally
Regional	Surviving globally	Surviving regionally
Local	Surviving regionally	Surviving locally

We fitted the model using the Nimble package version 0.12.2 (*62*) in R and ran six chains over 50,000 iterations, with a burn-in period of 1000 iterations and a thinning interval of 5. We used the reversible-jump MCMC method to highlight the most significant predictors in each regression (*30*). The reversible jump method associates each model coefficient with a binary inclusion parameter (0: excluded and 1: included). The model estimates the likely value of this model parameter over all model iterations and provides an estimate of the inclusion probability. The inclusion probability is the proportion of times a variable is included in the model across all MCMC iterations. It is a metric of the evidential support for a given predictor. We considered variables significant if they had an inclusion probability of 0.5 or higher, meaning they were more likely to be included rather than excluded by the model.

We accounted for the uncertainty surrounding missing trait values by, for each missing trait value, providing all 1000 imputed trait values as a data input to the model. On the basis these 1000 imputed values, the model estimated the underlying distribution for each missing trait value and each MCMC iteration and randomly drew values from that distribution. This meant that our total model estimated its parameters using different potential trait values, but that all three regressions used identical trait values during each MCMC iteration, keeping their results comparable at each step.

All variables were included at all scales, except for site variables. Site variables were included at the local scale only to account for their known effects on extinction risk rather than to test their consistency across scales. For an overview of all variables included in each regression, see tables S1 to S6. We scaled all predictor variables prior to the analysis, by centering the data around its mean and dividing it by its standard deviation. We used uniform distribution ranging from 0 to 1 as the priors for the reversible jump parameters. We used a logistic distribution with location 0 and scale 1 as the prior for the coefficient parameters.

We calculated the median of the posterior distribution of each coefficient, the 95% Bayesian credible interval, and inclusion probability. We made sure the model parameter estimates converged, by visually appraising the convergence plots and by checking the Gelman-Rubin statistic for each parameter ensuring they remained below 1.1.

## Supplementary Materials

### The PDF file includes:

Clarification of the Trait Metadata Models with Phylogenetic Correction Figs. S1 to S12 Tables S1 to S7 Legends for data S1 to S6 Legend for vignette S1

### Other Supplementary Material for this manuscript includes the following:

Data S1 to S6

Vignette S1
